# Anoctamin 6 Regulates C2C12 Myoblast Proliferation

**DOI:** 10.1371/journal.pone.0092749

**Published:** 2014-03-24

**Authors:** Piming Zhao, Audrey Torcaso, Andrew Mariano, Li Xu, Sadia Mohsin, Lixia Zhao, Renzhi Han

**Affiliations:** Department of Cell and Molecular Physiology, Loyola University Chicago Health Science Division, Maywood, Illinois, United States of America; Tohoku University, Japan

## Abstract

Anoctamin 6 (*Ano6*) belongs to a conserved gene family (TMEM16) predicted to code for eight transmembrane proteins with putative Ca^2+^-activated chloride channel (CaCC) activity. Recent work revealed that disruption of *ANO6* leads to a blood coagulation defect and impaired skeletal development. However, its function in skeletal muscle cells remains to be determined. By using a RNA interference mediated (RNAi) loss-of-function approach, we show that *Ano6* regulates C2C12 myoblast proliferation. *Ano6* is highly expressed in C2C12 myoblasts and its expression decreases upon differentiation. Knocking down *Ano6* significantly reduces C2C12 myoblast proliferation but has minimal effect on differentiation. *Ano6* deficiency significantly reduces ERK/AKT phosphorylation, which has been shown to be involved in regulation of cancer cell proliferation by another Anoctamin member. Taken together, our data demonstrate for the first time that *Ano6* plays an essential role in C2C12 myoblast proliferation, likely via regulating the ERK/AKT signaling pathway.

## Introduction

The anoctamin family (also referred to as TMEM16) is comprised of 10 proteins, each possessing eight transmembrane domains and cytosolic amino- and carboxyl-termini [1], [2]. They show distinct but overlapping expression patterns in a variety of cell types and tissues during development [3], [4].

Recently, several members of the anoctamin family have been identified as CaCCs in several tissues and cell lines [4], [5], [6], [7], [8], [9], [10]. Studies on the electrophysiological properties of Anoctamin family members revealed that most of them including Ano6 can confer chloride conductance in cell culture [9]. Ano6 has been characterized as an outwardly rectifying chloride channel [11] with delayed activation by calcium [12]. In addition to its potential function as a chloride channel, Ano6 has recently been identified as a calcium-activated cation channel that regulates Ca^2+^-dependent phosphatidylserine (PS) scrambling from the interior to exterior leaflet of the plasma membrane in blood cells [5]. *Ano6* has been shown to be expressed in mouse skeletal muscle by real-time RT-PCR analysis, along with moderate expression of several other ubiquitously expressed anoctamins and lower levels of *Ano1* and *4* [3], [4]. However, the cellular functions of Ano6 in skeletal muscle have not been determined.

In the present study, we sought to investigate the functions of anoctamins during myogenesis, a highly ordered process occurring during postnatal growth and the regeneration of skeletal muscle in response to injuries. Initial RT-PCR analysis demonstrated an intriguing pattern of *Ano6* expression during myoblast differentiation and mouse muscle development, therefore leading to further investigation. By using a RNAi-mediated loss-of-function approach, we examined the effects of Ano6 deficiency on myoblast proliferation and differentiation in a widely used murine muscle cell line, C2C12.

## Results

### Expression of Ano6 during C2C12 myoblast differentiation and in mouse skeletal muscle

**Figure 1:**
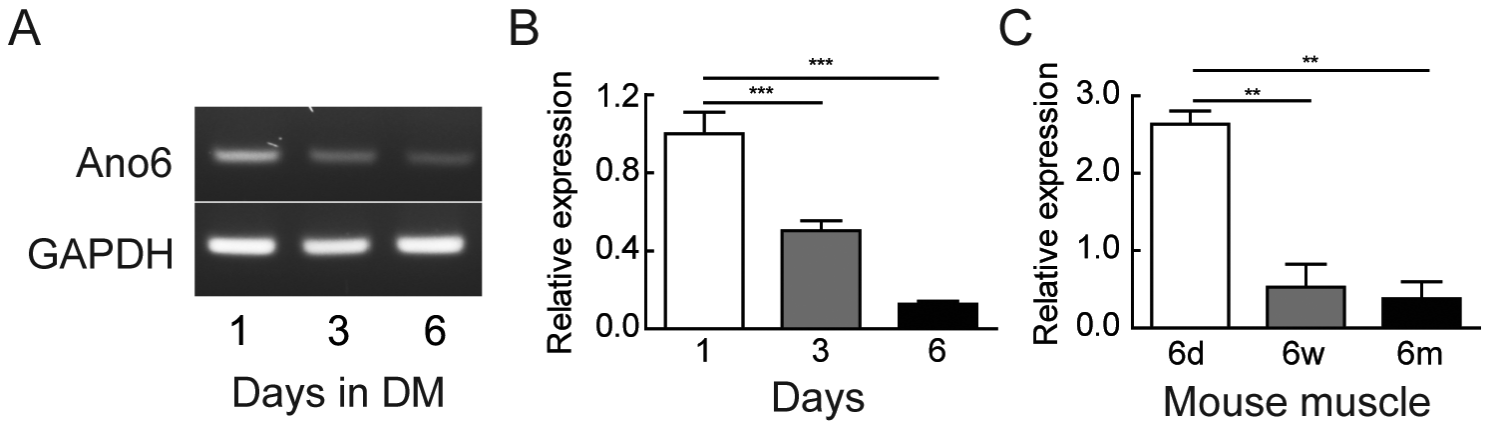
Expression of Ano6 in C2C12 muscle cells and mouse skeletal muscle. (A) Semi-quantitative RT-PCR analysis of Ano6 and GAPDH expression in C2C12 cells during differentiation. (B) Relative expression of Ano6 (normalized to GAPDH) examined by qRT-PCR in C2C12 cells during differentiation. (C) Relative expression of Ano6 (normalized to GAPDH) examined by qRT-PCR in the quadriceps muscles of mice at 6 days, 6 weeks and 6 months of age. **p<0.01; ***p<0.001.

*Ano6* expression was studied in C2C12 mouse muscle cell line by RT-PCR to investigate its possible role in myogenesis. *Ano6* expression declines from day 1 to day 6 in C2C12 cells upon differentiation (Fig. 1A). Concurrently, a 2-fold decline in *Ano6* expression was observed from day 1 to day 3 and a 8.3-fold decrease to day 6 (Fig. 1B; *p*<0.05) by quantitative real-time RT-PCR analysis, suggesting that Ano6 may play a role in myoblast maintenance.

To test whether a similar expression pattern exists in mouse skeletal muscle, we examined the expression of *Ano6* in young and adult skeletal muscle of wild-type mice. *Ano6* in skeletal muscle of 6-day-old pups was higher and decreased significantly by 5.2- fold at 6-week-old and by 8.6-fold in 6-month-old muscles as compared to 6 days old pups (Fig. 1C; *p*<0.001). This expression pattern in mouse skeletal muscle is consistent with that observed in differentiating C2C12 myocytes *in vitro*.

### Effect of Ano6 knockdown on C2C12 myoblast proliferation

**Figure 2:**
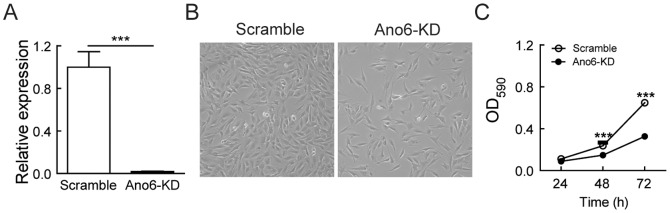
Effects of *Ano6*-KD on the proliferation of C2C12 myoblasts. (A) Relative expression of Ano6 (normalized to GAPDH) examined by quantitative RT-PCR in C2C12 stable cells lines (Scramble [shSCR], *Ano6*-KD). (B) Representative photographs of stable C2C12 cell lines expressing either a Scramble shRNA or the shRNA targeting *Ano6*-KD 48 hours post plating. (C) Quantitative analysis of C2C12 myoblast proliferation using the MTT assay. Scale bar  =  150 μm. ***p<0.001.

To study the role of Ano6 in C2C12 myoblast proliferation, we generated and screened seven knockdown (KD) constructs with their expression driven by the human U6 promoter. One of the KD constructs designated as shRNA-1989 showed the highest level of KD efficiency (∼85%) in a stable HEK293 cell line expressing mouse *Ano6* tagged with mCherry (Fig. S1). We then constructed lentiviral vectors expressing either a scramble shRNA or the shRNA-1989 and singly transduced C2C12 cells to create stable cell lines with these lentiviruses. The expression of *Ano6* in the stable *Ano6*-knockdown (*Ano6*-KD) line was reduced by 90% as compared to the Scramble line (Fig. 2A). The Scramble and *Ano6*-KD C2C12 cell lines were plated at the same initial densities and were observed for two days in growth media. Microscope images taken at 48 hours post plating revealed differences in their proliferation capacity (Fig. 2B). *Ano6*-KD C2C12 cells reached only about 40-50% confluence, while the Scramble C2C12 cells reached almost complete confluence (Fig. 2B). Consistently, the metabolic rate as measured by the MTT assay in the Ano6 KD C2C12 cells decreased when compared with Scramble controls. A 1.6-fold decrease in the metabolic rate was observed after 48 hours (Fig. 2C; *p*<0.001) and a 3.5 -fold decrease after 72 hours in growth media when compared with 24 hours (Fig. 2C; *p*<0.001). These data suggest that Ano6 is essential for normal maintenance of C2C12 myoblasts.

### Altered ERK/AKT signaling pathway in Ano6-KD C2C12 myoblasts

**Figure 3:**
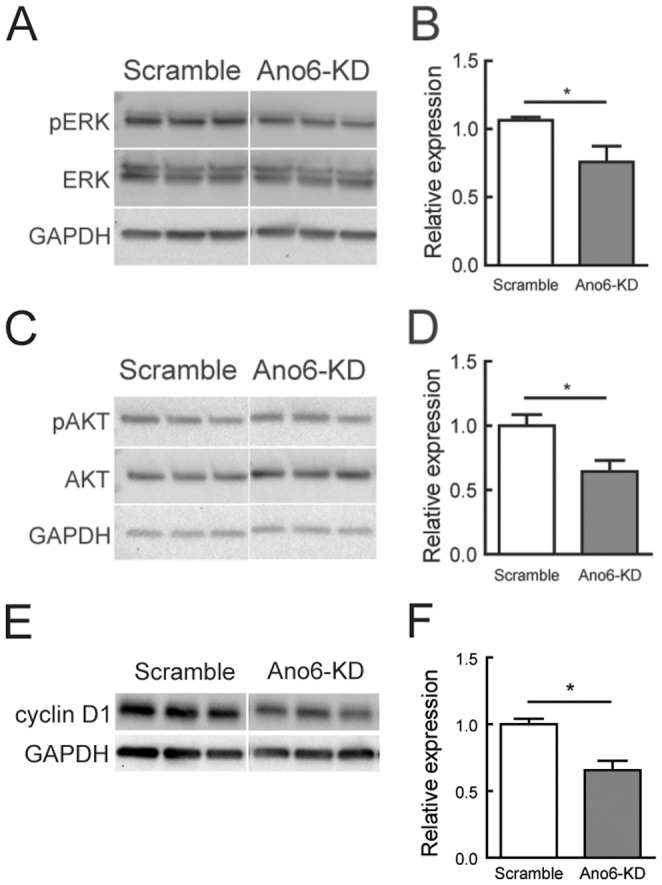
Altered ERK/AKT signaling in *Ano6*-KD C2C12 myoblasts. (A) Western blotting analysis of ERK phosphorylation (pERK) and total ERK expression (ERK) in C2C12 myoblasts of different stable lines. Three independent experiments per cell line were loaded on the gel. (B) Normalized expression levels of ERK phosphorylation and total ERK by membrane densitometry. (C) Western blotting analysis of AKT phosphorylation (pAKT) and total AKT expression (AKT) in C2C12 myoblasts of different stable lines. Three independent experiments per cell line were loaded on the gel. (D) Normalized expression levels of AKT phosphorylation and total AKT by membrane densitometry. (E) Western blotting analysis of cyclin D1 in C2C12 myoblasts of different stable lines. (F) Normalized expression levels of cyclin D1 by membrane densitometry. Three independent experiments per cell line were loaded on the gel. GAPDH was used as a loading control. *p<0.05.

**Figure 4:**
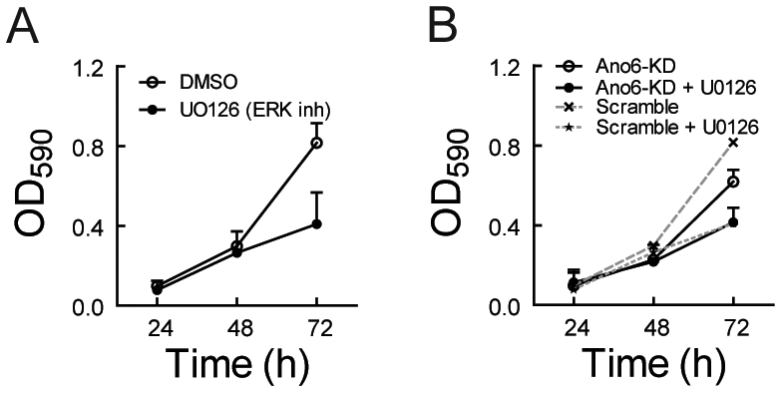
Effect of the ERK inhibitor UO126 on proliferation of control and *Ano6*-KD C2C12 myoblasts. (A) Proliferation analysis of control C2C12 cells treated by UO126 (10 μM) or the vehicle alone (DMSO) measured by MTT assay. (B) Proliferation analysis of *Ano6*-KD C2C12 cells treated by UO126 or the vehicle alone (DMSO) measured by MTT assay. Note that the dashed lines were re-plotted from panel A.

Recently, several groups reported that anoctamin 1 (Ano1) plays a role in cancer cell proliferation [13], [14], [15]; in breast cancer, it does so through the ERK/AKT signaling pathway [13]. We reasoned that Ano6 may regulate myoblast proliferation similarly via the ERK/AKT signaling pathway. Indeed, we observed that *Ano6*-KD significantly reduced ERK phosphorylation while the total ERK protein was not affected (Fig. 3A,B). In addition, *Ano6*-KD also affected levels of phosphorylated and total AKT levels (Fig. 3C,D). Cyclin D1, a downstream target of the ERK signaling pathway, plays an important role in cell cycle progression in C2C12 myoblasts [16]. Interestingly, cyclin D1 was also significantly attenuated by *Ano6*-KD (Fig. 3E,F). Thus, it is very likely that Ano6 regulates C2C12 proliferation by affecting the ERK/AKT signaling pathway. Consistent with this, pharmacological inhibition of ERK phosphorylation using UO126 [17] also significantly reduced the proliferation rate of C2C12 cells (Fig. 4A). Moreover, no additive effect of UO126 was observed on the proliferation rate when adding into *Ano6*-KD cells (Fig. 4B). These results suggest that Ano6 regulates the ERK/AKT signaling pathway, maintaining the proliferating status of C2C12 myoblasts.

### Effect of Ano6 deficiency on C2C12 myoblast differentiation

**Figure 5:**
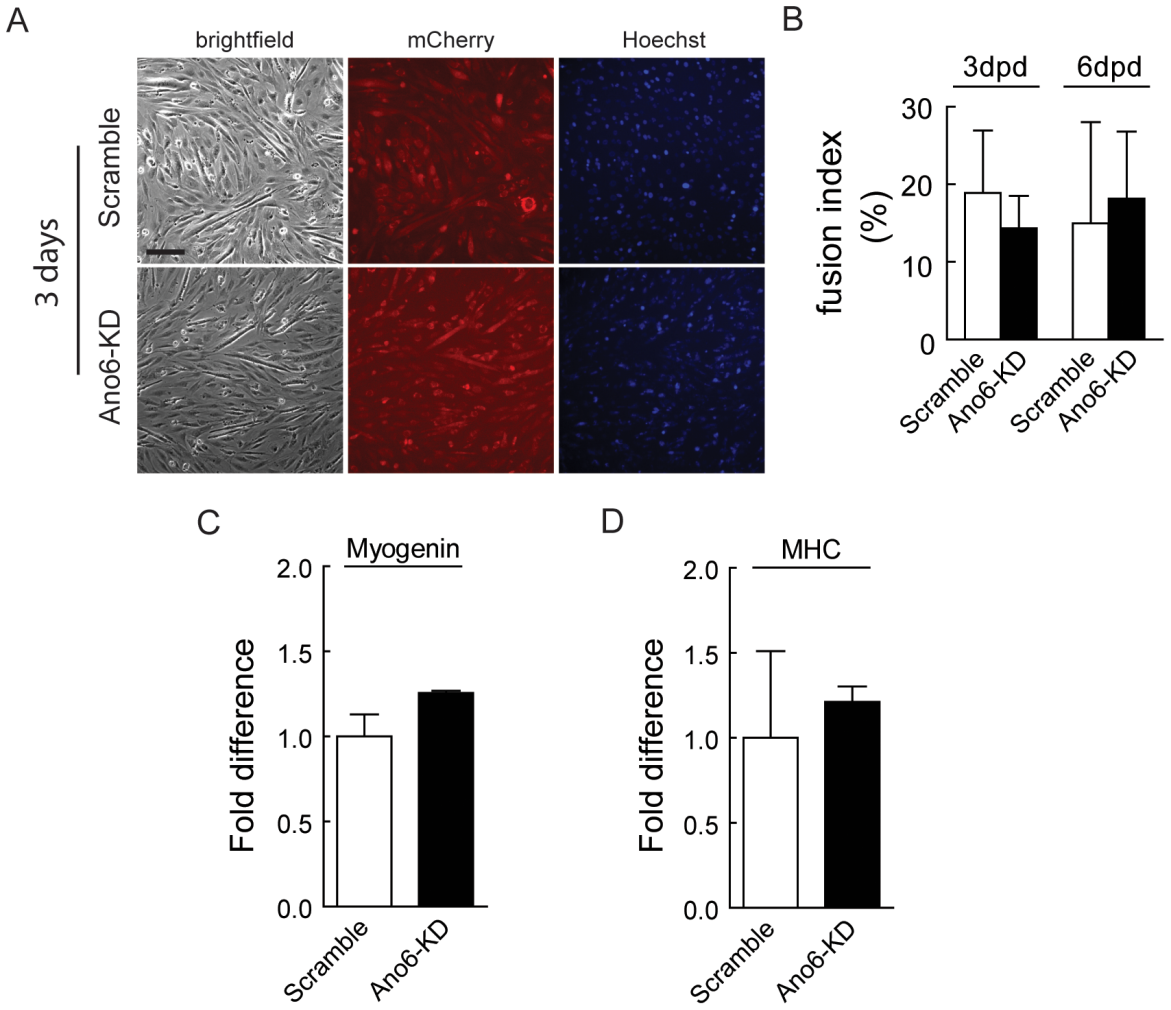
Representative micrographs of Scramble and *Ano6*-KD C2C12 myotubes. (A) Scramble and *Ano6*-KD stable cells, which also express mCherry, were imaged 3days after differentiation. (B) Fusion index of Scramble and *Ano6*-KD stable C2C12 cells after differentiation for 3 days. Scale bar  =  150 μm. (C,D) Fold difference of myogenin and myosin heavy chain (MHC) expression as examined by RT-PCR of C2C12 cells during differentiation.

Our data demonstrated that the expression of *Ano6* is decreased during differentiation. To test whether Ano6 plays a direct role in differentiation, we compared the *Ano6*-KD cell line with the Scramble control in their capacity to differentiate. The stable C2C12 cell lines were induced to differentiate by replacing 10% fetal bovine serum with 2% heat-inactivated horse serum. Knocking down *Ano6* did not significantly affect myoblast differentiation (Fig. 5A). To quantify such effect, the fusion index was calculated on day 3 and day 6 after differentiation. Knocking down *Ano6* had no significant effects on the fusion index (Fig. 5B). Consistently, molecular markers for myogenic differentiation including myogenin and myosin heavy chain were found to be expressed at the similar levels in both cell lines as examined by quantitative RT-PCR (Fig. 5C,D).

## Discussion

In the present study, we demonstrate that *Ano6* is highly expressed in undifferentiated myoblasts with peak expression during initial stages of myotube formation *in vitro*. Using a shRNA KD approach, we show that *Ano6*-KD C2C12 myoblasts exhibit reduced proliferation capacity. Our data demonstrate that Ano6 is required to maintain the proliferative status of myoblasts.

While little is known about the molecular and cellular functions of Ano6, much work has been done to characterize other anoctamins. The closely related Ano1 has been shown to be upregulated in many cancers and it possesses CaCC activity, which stimulates cell proliferation [3], [9], [13], [14], [15], [18]. In contrast to its role in stimulating cell proliferation, Ano1 has also been shown to inhibit angiotensin-2-mediated proliferation of basilar smooth muscle cells, suggesting that Ano1 plays different roles in different cell types [19]. This cell-type specific function appears to be also true for Ano6. Our findings suggest that Ano6 plays an important role in myoblast proliferation, whereas a previous study showed that Ano6 had no effect on osteoblast proliferation although its deficiency leads to reduced skeleton size and skeletal deformities [20]. Our data have also shown that *Ano6*-KD significantly attenuates ERK phosphorylation, which is implicated in the regulation of cancer cell proliferation by Ano1, suggesting that Ano6 is potentially involved in regulating myoblast proliferation through the ERK signaling pathway. Consistent with this, pharmacological inhibition of ERK phosphorylation also reduces the myoblast proliferation. Therefore, our data have revealed a novel regulator of myoblast proliferation.

Our data did not observe any significant effect of *Ano6* deficiency on myoblast differentiation or myogenic differentiation markers. In our experimental settings, we plated equal density of Scramble and *Ano6*-KD C2C12 cells for differentiation. Since *Ano6* deficiency impairs myoblast proliferation, it is conceivable that the differentiation program will be affected if the same amount of control and *Ano6*-KD cells were allowed to proliferate for several days before inducing differentiation. We speculate that Ano6 deficiency will lead to smaller muscles *in vivo*. Interestingly, it was previously reported that deletion of *Ano6* in mice results in reduced skeleton size and skeletal deformities although the authors did not focus on the skeletal muscle phenotype [20]. It is possible that the skeletal muscle in the *Ano6*-null mice may also be affected. Therefore, it would be interesting to study the effect of *in vivo Ano6* disruption on skeletal muscle during development and regeneration in response to injuries in the future.

Although complex, recent progress has illuminated some of the molecular and cellular functions of Ano6. Like other members of the anoctamin family, Ano6 has been shown to act as a relatively weak CaCC in the plasma membrane [3], [9], [18]. In addition, Ano6 has been shown to function as a Ca^2+^-activated cation channel that is required for PS scrambling in platelets during blood coagulation [21]. Indeed, mutations in *ANO6* have been identified in patients with Scott syndrome, a rare genetic bleeding disorder caused by a defect in PS scrambling in platelets [5]. The functional complexity of Ano6 is further exemplified by a recent study showing that calcium-activated Ano6-mediated phospholipid scrambling can occur independently of Ano6 ion currents [22]. At present, it is unknown whether Ano6 functions as a Ca^2+^-activated chloride or cation channel in myoblasts, and whether it plays a role in PS exposure in myoblasts. It is also not known whether any of these functions is related to the regulatory role of Ano6 in myoblast proliferation. It is possible that Ano6-mediated Ca^2+^-activated cation channel activity raises the intracellular Ca^2+^ concentrations, thereby causing the activation of ERK through Ca^2+^-regulated signaling cascades [23]. Evidence also exists to support a potential regulation of ERK by PS. For example, PS was shown to induce ERK activation in osteogenic differentiation of human mesenchymal stem cells [24]. In macrophages, the activation of ERK by PS is mediated by PS-specific receptor [25]. Finally, studies on Ano1 in cancer cells suggest that the chloride conductance appears to be a novel regulator of ERK signaling [26]. Mutation in the putative pore forming domain of Ano1 (K610A) abrogates its chloride conductance [18] and blocks its induction of ERK phosphorylation [26]. Similarly, the selective Ano1 inhibitor T16A(inh)-A01 [27], which inhibits CaCC currents, also reduces proliferation of interstitial cells of Cajal and a pancreatic cancer cell line CFPAC-1 [28]. These studies together favor an intriguing possibility that the CaCC currents through TMEM16 protein family may regulate cell proliferation by affecting ERK activation. However, the exact mechanism by which this occurs remains to be determined. Future investigations are required to fully understand the molecular and cellular functions of Ano6 in muscle tissue.

In summary, our present study revealed an important cellular function of Ano6, which regulates myoblast proliferation likely through the ERK/AKT signaling pathway.

## Materials and Methods

### Ethics Statement

All animal studies were reviewed and approved by the Institutional Animal Care and Use Committee of Loyola University Chicago (LU#202288 and LU#202769). Mice were maintained at Loyola University Medical Center Animal Facility in accordance with animal usage guidelines.

### Mice

Wild-type C57BL6/J mice were used in this study. All animal studies were reviewed and approved by the Institutional Animal Care and Use Committee of Loyola University Chicago Health Science Division.

### Cell culture

C2C12 mouse muscle cell line [29] was obtained from ATCC and used for lentivirus transduction at low passages (P4 to P6). C2C12 myoblasts were grown in DMEM (without Sodium Pyruvate) media supplemented with 10% FBS, 2 mM L-glutamine, and 1% penicillin/streptomycin. To induce differentiation of C2C12 myoblasts cultured in collagen type I-coated dish, the growth medium (GM) was changed to differentiation medium (DM) by replacing 10% FBS with 2% horse serum two days after plating when the cells reached 80-90% confluence. Lentivirus packaging cell line HEK 293T were grown in DMEM with 10% FBS, 2 mM L-glutamine and 1% penicillin/streptomycin.

### Plasmid construction, adenovirus and lentivirus generation

Ano6 (kindly provided by Dr. Criss Hartzell) was subcloned into pLVX-mCherry-C1 vector (Clontech) for making Ano6 lentiviruses. To generate Ano6 shRNA constructs, the following target sequences were selected:

A6sh567 (GCACATCAAACTCCCGCTAAA), A6sh713 (GGATGAATGATTTCTACATCC), A6sh770 (GCCGCATTGTTTATTTCATCC), A6sh960 (GGCTCACCCTCGGAGTATATA), A6sh1007 (GGAAGTATTACGGCGAGAAGA), A6sh1096 (GCCTGCTTCCTCTATGGATAT), A6sh1767 (GACCCAGACGGATTATGAGAA), A6sh1989 (GGCAATCTGGAACAACATACA), and shSCR (AGGAGTTCGTTCGCTCTCC) for Scramble control shRNA. The shRNA oligonucleotides were synthesized to contain the sense strand of target sequences, short spacer (AACG or TTCAAGAGA) and the reverse complement sequences followed by five thymidines as an RNA polymerase III transcriptional stop signal. Oligos were annealed and cloned into the Mlu1 and HindIII sites in pRNAT-H1.1/Adeno shuttle vector and transferred into the adenovirus plasmid AdEasy-1 by homologous recombination. Adenovirus was generated using the AdEasy-1 Adenovirus system (Agilent Technologies, La Jolla, CA) according to the manufacturer's manual, followed by purification by ultracentrifugation. For lentivirus shRNA construction, pLKO.1-Puro was obtained from Addgene (Addgene plasmid #8453). Puromycin cassette was replaced by hygromycin to obtain pLKO.1-hygro. To construct pLKO.1-mCherry-Puro, puromycin cassette from pLKO.1-Puro was replaced by mCherry-2A-puromycin. Oligonucleotides were annealed and cloned into each pLKO.1 version vectors with AgeI/EcoRI sites. Lentiviral particles were produced in 293T cells by co-transfection of pLKO.1 with pCMV-dR8.2 dvpr (Addgene plasmid #8455) and pCMV-VSVG (Addgene plasmid #8454) and purified by ultracentrifugation.

### Adenovirus and lentivirus transduction

Ano6 and scamble shRNA adenoviruses were used at 100 multiplicity of infection (MOI) to infect C2C12 myoblasts at 50–60% confluence. The GM was changed to DM 24 hours post transduction. After 3 days in DM, the C2C12 myotubes were harvested to analyze the knockdown (KD) efficiency by real-time quantitative RT-PCR. To initially screen the KD efficiency of various Ano6 shRNAs, Ano6-mcherry lentivirus was used to transduce HEK293 cells to obtain stable Ano6-expressing cell line. Ano6 shRNA lentivirus were used to infect Ano6 over-expression 293T cell line, two day after transduction cell was harvested for analyzing *Ano6*-KD efficiency. Ano6 shRNA lentiviruses were used to transduce low passage C2C12 cells (passage 4 to 6) to generate Ano6 stable KD C2C12 cell lines. Two days after lentivirus transduction, the cells were selected for about a week with either 1 μg/ml puromycin or 200 μg/ml hygromycin in the culture media. The stable cell lines were passaged and maintained in normal grow media without puromycin or hygromycin at least two more passages to avoid any side effects of antibiotics on C2C12 differentiation.

### RNA isolation, RT-PCR and qRT-PCR

Total RNA was extracted from mouse tissues and C2C12 cells by using Trizol reagent (Life Technologies, Carlsbad, CA). Total RNA was pre-treated with an RNase-free DNase and 4 μg of treated RNA was used as template for first-strand cDNA synthesis by using the SuperScript III First-Strand Synthesis System (Life Technologies, Carlsbad, CA). Aliquots of the RT product were used for regular and quantitative RT-PCR. Quantitative RT-PCR (qPCR) was performed using GoTaq qPCR Master Mix (Promega, Madison, WI) in CFX96 Touch Real-Time PCR Detection System (Bio-Rad, Hercules, CA) and normalized to glyceraldehyde 3-phosphate dehydrogenase (GAPDH). The primers used in this study include: Ano6-F, 5′ CCGCCTGGTGTATTATTGGTCTT 3′; Ano6-R, TGTTCTTGAAGTCCGTGATGTTGA 3′;; GAPDH-F, 5′ ACCTGCCAAGTATGATGA 3′; GAPDH-R, 5′ GGAGTTGCTGTTGAAGTC 3′.

### Cell proliferation assay

Cell proliferation was measured by Thiazolyl Blue Tetrazolium Bromide (MTT;Sigma, St. Louis, MO) method. Scramble and *Ano6*-KD C2C12 myoblasts were plated at 3×10^3^ cells per well in 96-well plates and assayed at 24, 48 and 72 hours post plating. When indicated, 10 μM UO126 was applied 24 hours post plating. GM were carefully removed at indicated time and replaced with 0.5 mg/ml MTT in DMEM media (without phenol red) for 3.5 hours. After removing MTT solution, cells were lysed with 4 mM HCl and 0.1% NP-40 in isopropanol and optical density was measured at 590 nm using an absorbance microplate reader (BioTek Epoch, Winooski, VT).

### Microscopy, nuclei staining, and calculation of fusion index

Ano6 shRNA C2C12 myotubes with mCherry were imaged by NIKON ECLIPSE Ti epi-fluorescence microscope. Cell nuclei were stained with H33258 for 30 minutes. Myotube nuclei were counted using Image J. Fusion index was calculated by counting the percentage of the nuclei within C2C12 myotubes (defined as containing >2 nuclei) out of total nuclei within the entire photographed field. An average of 3 randomly-encountered photographed fields per biological replicate were used.

### Immunoblotting

C2C12 myoblasts and myotubes were lysed with RIPA buffer (150 mM NaCl, 1 mM EDTA, 1% NP-40, 0.1% SDS, 20 mM Tris-HCl pH 8.0) with 1× Protease Inhibitor Cocktail (Cell Signaling Technology, Danvers, MA). Proteins were separated on 4%–15% SDS-PAGE, transferred onto PVDF membranes, and blotted as previously described [30]. Antibodies AKT, Phospho AKT, ERK and phosphor ERK was purchased from Cell Signaling Technology, Danvers, MA). GAPDH antibody (EMD Millipore, Billerica, MA) was used as a loading control.

### Statistical analysis

All data were expressed as mean ± standard deviation (SD). Statistical differences were determined by unpaired Student's *t*-test for two groups and one-way ANOVA with Bonferroni's post-tests for multiple groups using Prism 5.02 (Graphpad). The *p* values less than 0.05 were considered to be significant.

## Supporting Information

Figure S1 **Quantitative RT-PCR analysis of** ***Ano6*** **expression (normalized to GAPDH) in stable** ***Ano6*** **-mCherry-expressing HEK293 cells transduced with lentiviral particles expressing different shRNAs.**
(TIF)
